# A comparison of landmark methods and time-dependent ROC methods to evaluate the time-varying performance of prognostic markers for survival outcomes

**DOI:** 10.1186/s41512-019-0057-6

**Published:** 2019-07-25

**Authors:** Aasthaa Bansal, Patrick J. Heagerty

**Affiliations:** 1 The Comparative Health Outcomes, Policy, and Economics (CHOICE) Institute, University of Washington, H-375 Health Sciences Building, Campus Mail Stop 357630, Seattle, 98195 WA USA; 2 Department of Biostatistics, University of Washington, F-600 Health Sciences Building, Campus Mail Stop 357232, Seattle, 98195 WA USA

**Keywords:** Biomarkers, Hazard ratio, Medical decision-making, Prognosis, ROC curve

## Abstract

**Background:**

Prognostic markers use an individual’s characteristics at a given time to predict future disease events, with the ultimate goal of guiding medical decision-making. If an accurate prediction can be made, then a prognostic marker could be used clinically to identify those subjects at greatest risk for future adverse events and may be used to define populations appropriate for targeted therapeutic intervention. Often, a marker is measured at a single baseline time point such as disease diagnosis, and then used to guide decisions at multiple subsequent time points. However, the performance of candidate markers may vary over time as an individual’s underlying clinical status changes.

**Methods:**

We provide an overview and comparison of modern statistical methods for evaluating the time-varying accuracy of a baseline prognostic marker. We compare approaches that consider cumulative versus incident events. Additionally, we compare the common approach of using hazard ratios obtained from Cox proportional hazards regression to more recently developed approaches using time-dependent receiver operating characteristic (ROC) curves. The alternative statistical summaries are illustrated using a multiple myeloma study of candidate biomarkers.

**Results:**

We found that time-varying HRs, HR (*t*), using local linear estimation revealed time trends more clearly by directly estimating the association at each time point *t*, compared to landmark analyses, which averaged across time ≥ *t*. Comparing area under the ROC curve (AUC) summaries, there was close agreement between AUC ^*C*/*D*^(*t*,*t*+1) which defines cases cumulatively over 1-year intervals and AUC ^*I*/*D*^(*t*) which defines cases as incident events. HR (*t*) was more consistent with AUC ^*I*/*D*^(*t*), as estimation of these measures is localized at each time point.

**Conclusions:**

We compared alternative summaries for quantifying a prognostic marker’s time-varying performance. Although landmark-based predictions may be useful when patient predictions are needed at select times, a focus on incident events naturally facilitates evaluating trends in performance over time.

## Background

Effective clinical practice relies on informed decision-making, which is ideally guided by predictions of a patient’s future clinical health status under alternative treatment options. For example, a cancer patient who has previously undergone treatment but is predicted to be at high risk of disease recurrence may benefit from adjuvant therapy, whereas a patient at low risk of recurrence may be spared the side-effects of aggressive treatment. Predictions of future disease events can be made using an individual’s clinical characteristics, which serve as candidate prognostic markers for future onset or progression of disease. The term “prognostic marker” may refer to a single biomarker such as a specific serum protein measure, or to a composite score calculated as a combination of multiple risk factors. For example, multimarker recurrence risk scores have been developed and now impact clinical care [1]. A good prognostic marker effectively guides the choice and timing of therapeutic interventions, enabling timely action for those individuals at greatest risk of experiencing an adverse event.

Often, a marker measured at a single time point is used to make decisions at multiple time points in the future. For example, Harris et al. [11] review thirteen categories of breast cancer tumor biomarkers and comment on those that are recommended for use in practice, including estrogen receptor status, progesterone receptor status, and human epidermal growth factor receptor 2. However, any given marker may have predictive accuracy that varies over time. For instance, a marker may discriminate accurately between high-risk and low-risk populations shortly after baseline. However, 5 years after baseline, the same biomarker may not retain accuracy and therefore may not be useful for later clinical decisions. The goal of this manuscript is to overview modern statistical methods that address the two following questions: how can the prognostic potential of a biomarker be evaluated over time and how can different candidate markers be directly compared?

Fundamental to epidemiology and clinical research are the diagnostic concepts of sensitivity and specificity. Sensitivity is essentially a cross-sectional measure describing the probability of a positive test given that an individual is diseased. However, most disease states change over time and basic descriptive epidemiology clearly distinguishes between prevalent and incident disease cases. Only recently have statistical methods been developed that can generalize cross-sectional accuracy concepts for application to the time-varying nature of disease states, and corresponding definitions of sensitivity and specificity have been proposed for both prevalent and incident case definitions [12, 13]. These new concepts and associated statistical methods are central to the evaluation of the time-varying performance of any potential prognostic marker.

When prognostic markers are studied using event-time data from prospective studies, the outcome of interest is the time until some key clinical event, such as death or disease progression. At a fixed time point, the risk set, or the set of individuals still at risk for the event, may be partitioned into *cases* and *controls*. Cases are individuals who experience the outcome, whereas controls are those individuals who do not (yet) experience the event. Moreover, cases may be defined as *incident* cases or *cumulative* cases. As the terms suggest, incident cases are individuals who experience the event *at* the given time point, whereas cumulative cases are those individuals experiencing events that are observed over a specified duration of time. Controls are generally defined as the remaining event-free subjects, and the performance of a prognostic marker is determined by how accurately it distinguishes between appropriately defined cases and controls. Note that as time progresses and events accumulate, the sets of cases and controls change, and so too may a marker’s ability to distinguish cases and controls.

A number of existing statistical methods build upon these basic ideas for the proper characterization of a marker’s prognostic accuracy; however, knowledge of these methods and the tools available to implement them remains limited. As a result, although numerous studies seek to develop prognostic markers across a range of disease settings, such studies often perform limited evaluation of time-varying marker utility.

Our goal in this paper is to demonstrate the use of modern statistical methods for properly characterizing the time-varying performance of a prognostic marker. In the “Methods” section, we review standard summaries that are typically used with event-time data in order to characterize the association between a marker and survival. Common summaries do not take into account the potential time-varying performance of markers. We introduce and discuss four different statistical summaries that characterize the time-varying prognostic ability of a marker. In the “Results” section, we illustrate these approaches using a multiple myeloma dataset. In the “Discussion” section, we include a summary of the results, comparison of the approaches, and some suggestions for future development. Finally, we close with some practical recommendations in the “Conclusions” section.

### Motivating example

Bargolie et al. [3] describe a prospective randomized trial that compared alternative treatments for multiple myeloma. Secondary analysis focused on select biomarkers measured at baseline, including albumin, creatinine, and serum beta-2-microglobulin. The primary goal of biomarker evaluation was to determine whether different markers were more prognostic at different times during follow-up. Specifically, a steep decline in survival was apparent during early follow-up and it was hypothesized that select markers may be prognostic during this period while others may be prognostic at later times. Bargolie et al. [4] used “landmark” survival analysis methods to investigate their hypothesis. We review landmark methods and suggest alternative methods that can focus on the time-varying evaluation and comparison of candidate biomarkers. We present a detailed comparative analysis of the myeloma study data.

## Methods

### Standard summaries

Time to event or survival data are typically obtained from prospective studies where a continuous follow-up time is observed for each participant and follow-up may end either due to occurrence of the event of interest or due to censoring. Censoring is a common issue in such data, and ignoring it can lead to biased assessments of a marker’s performance. Therefore, appropriate evaluation of a prognostic marker usually requires methods that are suitable for censored survival data. By convention, we assume that larger marker values are indicative of a poorer prognosis. When the opposite is true for a particular marker such that smaller values are linked to poorer prognosis, we transform the marker to fit the convention.

A common semiparametric approach to summarizing the association between a marker and survival is to fit a Cox proportional hazards regression model, which assumes the following form [6]:

1 $$\begin{array}{@{}rcl@{}} {\lambda(t|\mathbf{x}) = \lambda_{0}(t) exp (\Sigma_{j} \beta_{j} x_{j})} \end{array} $$

where *λ*(*t*|**x**) is the instantaneous rate of an event at time *t*, specified as a function of a set of covariates **x**. The parameter *λ*_0_(*t*) represents the baseline hazard function, and *β*_*j*_ is the regression coefficient or log hazard ratio corresponding to covariate *x*_*j*_. In assessing the association of a single marker *M* with failure, we simplify (1) to

2 $$\begin{array}{@{}rcl@{}}  \lambda(t|M) = \lambda_{0}(t) exp(\beta M), \end{array} $$

where *exp*(*β*) is the hazard ratio corresponding to marker *M*. The parameter *β* is equal to the logarithm of the instantaneous relative risk or multiplicative increase in the hazard of an event for a one-unit increase in *M* and measures the association between the marker and survival. While regression methods can assess the strength of association, they do not directly characterize the potential ability of the marker to separate cases and controls, nor do they directly measure the potential for the marker to accurately guide medical decisions.

A common approach to showing the ability of a marker to separate cases and controls is to display estimates of survival curves for different subgroups of patients grouped by their marker values. Frequently, non-parametric survival estimates are obtained using Kaplan-Meier (K-M) curves [14] for patients stratified on tertiles or quartiles of the marker. A formal method for comparing K-M survival curves is the log-rank test [17]. Graphically, the more separated the K-M curves, the stronger the association of the marker with survival, and implicitly, the marker has a greater ability to separate high-risk subjects from low-risk subjects.

These two standard approaches can be used to summarize association, but when scientific interest lies in characterizing the time-varying performance of a marker as a potential guide to decision-making, then alternative measures are warranted. One approach is to consider a marker-survival concordance index [10], while another recent approach is to define and estimate time-dependent error rates that extend the fundamental concepts of sensitivity and specificity to survival outcomes.

### Time-varying hazard ratios

In this section, we present two approaches that generalize Cox regression to allow hazard ratios to change with time.

#### Using a changing “baseline time”

**Fig. 1:**
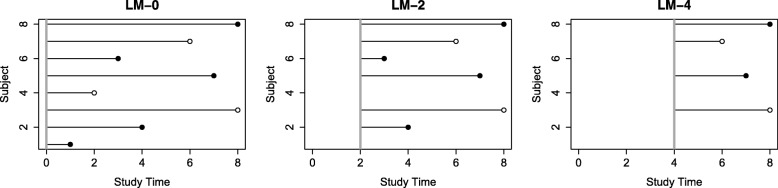
An illustration of landmark analysis. Solid circles represent events, and hollow circles represent censored subjects. For each landmark time point, subjects still alive then are used for analysis. The solid vertical line represents the landmark analysis cutoff time point

Landmark analysis [25] can be described as taking a sequence of follow-up evaluations conditional on survival to select “landmark” times. Specifically, a small number of index time points are chosen and survival analysis is done on only those subjects who remain event-free at the specified index times and for follow-up beyond the index times. Figure 1 illustrates the landmark idea for a series of time points: baseline, 2 years, and 4 years. In this approach, a Cox proportional hazards model would be fit on the subset of remaining subjects at each landmark time point, and a series of hazard ratios would be obtained for follow-up beyond the different time points. A time-varying association would be indicated by a change in hazard ratios across the landmark analysis times. Because Cox regression is a widely used tool for survival analysis and is available in all standard statistical software packages, the landmark approach is straightforward to conduct since it only requires that the data are subset to survival beyond the landmark time in order to perform the analyses. However, the interpretation of landmark analysis results is subtle because each landmark analysis returns a hazard ratio estimate that is interpreted as the *average* hazard ratio over the restricted time period from the landmark time (i.e., new baseline) to the end of the follow-up, (*t*_*j*_,*T*_final_). We denote these hazard ratios as HR (*t*_*j*_,*T*_final_). For example, using 2 years as a landmark time produces a set of regression coefficient estimates which would then be interpreted as the average hazard ratio over the time period from 2 years until the end of follow-up (2,*T*_final_) and denoted as HR (2,*T*_final_). By conducting landmark analyses, we are summarizing the changes in the average hazard ratio over nested time intervals that move away from the original baseline and therefore can indirectly infer changes in the underlying association between the biomarker and the time-specific risk of death or hazard. For example, if the landmark HR (2,*T*_final_) is larger than HR (3,*T*_final_), then we can indirectly infer that the risk associated with the marker is larger between times 2 and 3 years than the average HR after 3 years.

The landmark approach has recently been used by others and is discussed in the book by van Houwelingen and Putter [25]. As described above, Barlogie et al. [4] found that the survival curve estimated from a multiple myeloma study had differently shaped segments and used this feature as motivation for an investigation of the time-varying prognostic performance of candidate markers measured in the study. They carried out landmark analyses to summarize different segments of follow-up with the eventual goal of determining which markers dominated each segment. Their choice of landmark time points was baseline, 3 years, 4 years, 5 years, and 7 years, a data-driven choice based on exponential curves that best fit the observed survival curve. They measured the time-varying associations of the different variables by fitting univariate Cox models for each marker at each landmark time point. The resulting hazard ratios and associated *p* values were used to gauge the relative prognostic abilities of the variables over time.

Landmark analysis is a useful descriptive tool but has some key limitations. First, a hazard ratio obtained in this manner is an average of the marker-disease association starting at the landmark time point until the end of the study period. However, if scientific interest lies in estimating the association *at* given time points and characterizing how that association varies over time, then landmark analysis only indirectly answers this question. If the association does indeed vary over time, then averaging across time points is expected to flatten time trends. We address this issue next, by considering an alternative approach that estimates a time-varying coefficient function which yields a time-specific hazard ratio.

#### Using time-dependent coefficients

As discussed above, the standard Cox regression model assumes proportional hazards over time. In other words, it assumes that the hazard ratio comparing the risk associated with marker value *M*=*m*+1 relative to *M*=*m* remains constant over time. In the previous section, we discussed an approach that sequentially applies this model to landmark analysis times and captures one form of time-varying association by fitting the model on nested subsets of data. A more direct way of characterizing time-varying associations is an extension of the Cox model that allows time-varying coefficients where associated hazard ratios vary as smooth functions of time. The time-varying coefficient Cox model assumes the following form:

$$\begin{array}{@{}rcl@{}} {\lambda(t|\mathbf{x}) = \lambda_{0}(t) exp \{ \Sigma_{j} \beta_{j}(t) x_{j} \}.} \end{array} $$

Local linear maximum partial likelihood is a non-parametric smoothing method for estimating time-varying hazard ratios [5]. The general idea behind this method is to use a locally weighted fitting at each time point *t* to estimate the corresponding coefficient *β*(*t*). Specifically, using the observed event times in a window around a given *t*, the coefficient function is approximated by a linear function or a first-order Taylor expansion, from which a partial likelihood estimate for the linear function can be obtained. The estimate of the smoothed coefficient function, *β*(*t*), at *t* is then simply the value of the estimated locally linear coefficient function at *t*. Details on estimation and inference were provided by Cai and Sun [5]. The local linear estimation technique has been implemented as the llCoxReg() function of the R statistical software package risksetROC, publicly available from The Comprehensive R Archive Network (CRAN).

The Cox model with time-dependent coefficients is typically used in a scenario where covariate effects change over time and is a natural approach to characterizing the time-specific association between a candidate marker and survival times. The advantage of using a time-varying coefficient is that it permits inference regarding the association between a marker and subjects who fail *at* each possible follow-up time *t*. In contrast to landmark methods where hazard ratios are interpreted as average associations over a select follow-up time period, the time-varying coefficient function, *β*(*t*), characterizes a time-specific rather than time-averaged strength of association. We denote a hazard ratio obtained using this approach as HR (*t*)= exp{*β*(*t*)}.

However, a potential issue with the use of landmark methods or general varying coefficient regression methods is that scientific motivation for evaluating a biomarker is often coupled with potential use of the biomarker to guide medical decision-making. For example, the primary purpose of a prognostic marker may be to predict patient outcomes so that targeted therapy can be directed to those subjects at greatest risk of progression or death. The hazard ratio and the corresponding *p* value are simply measures of association and do not directly answer the question of predictive performance. A hazard ratio that may be considered significant in studies of association does not necessarily translate to high prediction accuracy. More appropriate measures of accuracy would assess classification error rates, specifically generalizations of sensitivity and specificity [15, 21]. We consider time-dependent receiver operating characteristic (ROC) curves as alternate summaries. In the next section, we begin with some background on time-dependent ROC curves, followed by two specific approaches to the current problem of characterizing the time-varying performance of a prognostic marker.

### Time-dependent ROC curves to characterize time-varying prognostic performance

In this section, we present two approaches that focus on evaluating biomarker accuracy by developing generalizations of sensitivity and specificity in order to characterize changes over time in the potential of the marker to accurately classify incident cases and current controls.

The traditional classification problem is based on a simple binary outcome, typically the presence or absence of disease. In classifying individuals as having disease or not, a marker is prone to two types of error. The first error occurs when a diseased patient is incorrectly classified as not having disease; this leads to delays in treatment, while the disease continues to progress. The second error occurs when conversely, a non-diseased individual is classified as having disease; this subjects the individual to unnecessary emotional distress and risks from follow-up medical procedures. Investigators typically aim to minimize these two errors by using markers that have both high sensitivity and specificity.

The sensitivity of a marker is the probability that it is positive in the presence of disease, or the true positive rate (TPR). Specificity is the probability that the marker is negative in the absence of disease, or one minus the false positive rate (1 − FPR). More formally, let *D* denote disease, with *D*=1 indicating presence and *D*=0 indicating absence of disease (for cases and controls, respectively). By convention, larger marker values are assumed to be more indicative of disease. For a continuous marker *M* and a fixed threshold *c*, we define

$$\begin{array}{@{}rcl@{}} \text{sensitivity}(c) &=& P(M > c | D=1)\\ \text{specificity}(c) &=& P(M \leq c | D=0) \end{array} $$

The receiver operating characteristic (ROC) curve is a standard tool that uses a continuous marker’s sensitivity and specificity to summarize its potential classification accuracy [8, 9, 20, 24]. A series of binary splits of *M* for all possible values of the threshold *c* are obtained, and the corresponding values for sensitivity (or TPR) are plotted against 1 − specificity (or FPR) to create the ROC curve for *M*.

A good diagnostic marker performs with high sensitivity as well as high specificity. Therefore, a perfect marker has an ROC curve that goes through the point (FPR,TPR)=(0,1), for 100% sensitivity and 100% specificity. On the other hand, the 45 ^∘^ line represents the ROC curve for a marker that is completely independent of disease and is equally likely to classify both cases and controls as having disease. In practice, ROC curves typically fall somewhere in between these two extremes.

A marker’s classification accuracy is most commonly quantified using a single-number summary measure, the area under the ROC curve (AUC). The AUC also represents the probability that given a randomly chosen case and a randomly chosen control, the case has a higher marker value:

$$\text{AUC} = P(M_{i} > M_{j} | D_{i}=1, D_{j}=0). $$

An AUC of 0.5 indicates no discrimination between cases and controls, whereas an AUC of 1.0 indicates perfect discrimination.

These definitions are specific to binary outcomes. Implicit in the use of traditional diagnostic TPR and FPR are current-status definitions of disease. More generally, disease status changes with time and precise definitions are necessary to include event (disease) timing in definitions of prognostic errors rates. In the last decade, time-dependent ROC curve methods that extend concepts of sensitivity and specificity and characterize prognostic accuracy for survival outcomes have been proposed in the statistical literature and adopted in practice. We review two such time-dependent approaches, which draw upon alternative fundamental case definitions: prevalent or cumulative cases and incident cases.

#### Cumulative (prevalent) cases/dynamic controls

The standard definitions of sensitivity and specificity are based on the cross-sectional classification of subjects into one of two disease states. A natural extension to the survival context, where disease state is time dependent, is to simply dichotomize the outcome at a time of interest, *t*, and define cases as subjects who experience the event before time *t* and controls as those who remain event-free through time *t*. In other words, letting *T* denote survival time and *s* denote the start time of case ascertainment (often *s*=0), cumulative cases (*C*) may be defined as subjects with an event time prior to *t*, *T*_*i*_∈(*s*,*t*), and dynamic controls (*D*) as subjects who are event-free at time *t*, *T*_*i*_>*t*. Then, for a fixed threshold *c*, time-dependent definitions for sensitivity and specificity follow:

$$\begin{array}{@{}rcl@{}} \text{sensitivity}^{C}(c|\text{start}=s, \text{stop}=t) &=& P(M > c | T \geq s, T \leq t) \\ \text{specificity}^{D}(c|\text{start}=s, \text{stop}=t) &=& P(M \leq c | T \geq s, T > t). \end{array} $$

These definitions are equivalent to those presented originally by Heagerty et al. [12], who implicitly fixed *s*=0 and defined cases and controls simply as subjects with *T*≤*t* and *T*>*t*, respectively. Here, we use the more explicit notation of Zheng and Heagerty [27] to indicate the start time *s* of the interval over which cases accrue, so that *T*≤*t* is written as *T*∈(*s*=0,*t*).

For a fixed specificity ^*D*^(*c*|*s*,*t*)=1−*p* (where *p* represents a fixed FPR), the time-dependent ROC value is defined as the corresponding value of sensitivity ^*C*^(*c*|*s*,*t*), or ROC$^{C/D}_{t}(p)$. The superscript ^*C*/*D*^ denotes the use of cumulative cases and dynamic controls. The time-dependent AUC can be defined as

AUC ^*C*/*D*^(*s*,*t*)=*P*(*M*_*i*_>*M*_*j*_|*T*_*i*_≥*s*,*T*_*i*_≤*t*,*T*_*j*_≥*s*,*T*_*j*_>*t*).

Here, AUC ^*C*/*D*^(*s*,*t*) is the probability that given a random subject *i* who experiences an event before time *t* (case) and a random subject *j* who remains event-free through time *t* (control), subject *i* has a larger marker value than subject *j*, assuming both subjects are event-free at time *s*.

Note that in the special case where no censoring is observed, the above dichotomization at time *t* translates to evaluating the marker using binary vital status outcomes at any time *t*. However, when follow-up is incomplete, as is often the case, censoring can be handled using non-parametric estimation methods for ROC$^{C/D}_{s,t}(p)$ and AUC ^*C*/*D*^(*s*,*t*) proposed by Heagerty et al. [12]. These have been implemented in a publicly available R statistical software package called survivalROC.

The cumulative/dynamic ROC curve is appropriate as a tool for evaluating prognostic accuracy when scientific interest lies in using a marker measured at baseline to identify individuals who are at risk of an adverse event before time *t*, in order to guide the timing of therapeutic interventions. For example, in developing a screening program, an individual who is at high risk of developing disease within the next 5 years would be considered a good candidate for intensive screening, whereas an individual with a low predicted risk may forego such procedures. A second example that we mentioned earlier relates to disease progression where a cancer patient who is at high risk of recurrence within the next 3 years may benefit from adjuvant therapy, while a patient at low risk of recurrence may be spared the unnecessary side-effects of aggressive treatment.

**Fig. 2:**
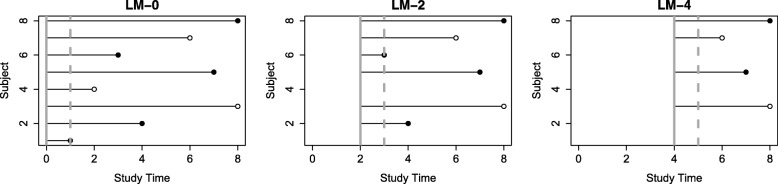
An illustration of ROC$_{t}^{C/D}$ mimicking landmark analysis. Solid circles represent events, and hollow circles represent censored subjects. For each landmark time point, subjects that remain alive are used for analysis. The solid vertical line represents this landmark analysis cutoff. The dashed vertical line represents the subsequent 1-year cutoff which is used to define cases versus controls

In motivation for this article, we seek to characterize the time-varying performance of candidate markers and the cumulative/dynamic ROC curve can be modified to provide a natural complement to the landmark analysis approach described above. We mimic landmark analysis by subsetting data at a sequence of landmark time points ${t^{L}_{1}}, {t^{L}_{2}}, \cdots, {t^{L}_{K}}$ to include only subjects with $T \geq {t^{L}_{k}}$, *k*=1,...,*K*. We then define cases cumulatively as subjects who have events over the following 1-year span, $T \in \left ({t^{L}_{k}}, {t^{L}_{k}} + 1\right)$, and controls such that $T > {t^{L}_{k}} + 1$. Figure 2 illustrates this idea.

Note that the superscript *L* is used to distinguish the time points described in this modified approach from those in a standard cumulative/dynamic ROC curve. Typically, time point *t* is used to define the endpoint of a window which begins at time point *s*=0 or baseline, such that cases have *T*∈(0,*t*) and controls have *T*>*t*. In contrast, in the modified approach, we re-define the case accumulation window so that $s = {t^{L}_{k}}$ and $t = {t^{L}_{k}} + 1$. Specifically, cases are defined such that they accrue in the window $T \in \left ({t^{L}_{k}}, {t^{L}_{k}} + 1\right)$ and controls such that they remain event-free, $T > {t^{L}_{k}} + 1$. The key utility of the cumulative/dynamic ROC approach is to generalize standard classification accuracy concepts to allow consideration of time. A basic formulation simply considers cumulative or prevalent cases that are observed during a well-defined follow-up period.

#### Time-dependent ROC curves: incident cases/dynamic controls

The cumulative/dynamic ROC curve discussed above uses a baseline or a landmark starting time point and a future follow-up time point *t* to define cases. However, survival analysis using Cox regression is based on the fundamental concept of a risk set: a risk set at time *t* is the case experiencing an event at time *t*, and the additional individuals who are under study (alive) but do not yet experience the clinical event. Extension of binary classification error concepts to risk sets leads naturally to adopting an incident (*I*) case definition where subjects who experience an event *at* time *t* or have survival time *T*_*i*_=*t* are the time-specific cases of interest. As before, dynamic controls (*D*) can be compared to incident cases and are subjects with *T*_*i*_>*t*. In this scenario, time-dependent definitions for sensitivity and specificity are:

$$\begin{array}{@{}rcl@{}} \text{sensitivity}^{I}(c|t) &=& P(M > c | T = t) \\ \text{specificity}^{D}(c|t) &=& P(M \leq c | T > t). \end{array} $$

Here, it follows that for a fixed specificity ^*D*^(*c*|*t*)=1−*p*, the time-dependent ROC value is defined as the corresponding value of sensitivity ^*I*^(*c*|*t*), or ROC$^{I/D}_{t}(p)$. Here, the superscript ^*I*/*D*^ denotes the use of incident cases and dynamic controls. The time-dependent AUC can then be defined as

$${\text{AUC}^{I/D}(t) = P(M_{i} > M_{j} | T_{i} = t, T_{j} > t)} $$

and has an analogous interpretation to AUC ^*C*/*D*^(*t*) above. In this setting, marker performance over time may be summarized using a global summary called the survival concordance index (C-index):

$${C = P(M_{i} > M_{j} | T_{i} < T_{j})} $$

The C-index is interpreted as the probability that the predictions for a random pair of subjects are concordant with their outcomes. In other words, it represents the probability that the subject who died at an earlier time had a larger marker value. The C-index can also be expressed as a weighted average of time-specific AUCs [13] and is therefore easy to estimate.

Semiparametric estimation methods based on the Cox model have been proposed for ROC$^{I/D}_{t}(p)$ and AUC ^*I*/*D*^(*t*) [13]. These have been implemented in a publicly available R package called risksetROC. Additionally, a non-parametric rank-based approach for the estimation of AUC ^*I*/*D*^(*t*) has been proposed by Saha-Chaudhuri and Heagerty [23]. The basic idea behind the rank-based approach is to compute for each risk set the binary concordance statistic using only the individual case and associated risk set controls. Here, the time-specific case is evaluated in terms of the number of risk set controls who have a smaller marker value. A perfect marker would have the case value greater than 100% of risk set controls. Specifically, for a fixed time point *t*, we calculate a percentile for each case in the risk set relative to the controls in the risk set. The mean percentile at time *t* is calculated as the mean of the percentiles for all cases in a window around *t*. The summary curve, AUC (*t*), is then estimated as the local average of case percentiles. The non-parametric approach provides both a simple description for marker performance within each risk set, and by smoothing these points, a final summary curve over time characterizes time-dependent accuracy.

The incident/dynamic ROC curve is particularly appropriate for evaluating the performance of a marker measured at baseline or at multiple time points in a scenario which requires therapeutic decisions to be made at a sequence of time points. For example, in an organ transplantation setting, interest lies in identifying patients who are at higher risk of death in the near future, so that they may be given priority for limited donor organs. The recipient decision may be made at multiple time points as donor organs become available, but is applicable to those subjects who still remain at risk at those times.

The idea of evaluating the performance of a marker at a sequence of time points lends itself naturally to evaluating time-varying performance just as Cox regression allows risk modeling as a function of time. In the previous section, we described a modified version of the cumulative/dynamic ROC curve, which used landmark analysis with cases defined cumulatively over 1-year windows. The advantage of using the incident/dynamic ROC curve is that it uses a finer timescale. For time point *t*, instead of defining cases cumulatively over the following year, an incident approach focuses on cases that occur *at* time *t*. Additionally, AUC ^*I*/*D*^(*t*) can be easily summarized across time using the C-index as shown by Heagerty and Zheng [13].

### Illustration of methods using multiple myeloma dataset

We illustrate the methods discussed above on a motivating dataset from a multiple myeloma treatment study.

#### Study description

The data that we analyze are from a prospective randomized trial that compared high-dose chemoradiotherapy to standard chemotherapy among subjects with multiple myeloma (MM). The trial was conducted by three North American Cooperative Groups (Southwest Oncology Group, Eastern Cooperative Oncology Group, and Cancer and Leukemia Group B), which recruited subjects who were untreated for and symptomatic of MM, were ≤ 70 years old, and had Zubrod performance status of 0 to 2 (performance status of 3 to 4 resulting from myeloma-related bone disease was acceptable). Further details about the study can be found in the original article [3].

For our analysis, 775 patients aged 25–70 were available, with a median follow-up of 8.2 years and median survival of 4.0 years. Survival was similar in both study arms, and therefore, subjects were pooled together for prognostic marker analysis.

A number of baseline variables were measured, of which 8 were continuous and therefore considered by us as prognostic marker candidates. These were age, albumin, calcium, creatinine, hemoglobin, lactic hydrogenase (LDH), platelet count, and serum beta-2-microglobulin (SB2M). Barlogie et al. [4] used the same dataset to carry out the landmark analysis described above.

#### Analytic approach

To estimate hazard ratios, we log-transformed variables with skewed distributions; these included albumin, creatinine, LDH, and SB2M. Additionally, recall that a hazard ratio represents the increase in risk associated with a one-unit increase in the marker value. Since the markers were measured on different scales, we made the hazard ratios for different markers comparable by standardizing the markers. Note that log-transformation and standardization are done to facilitate marker comparison when using regression methods but are not necessary for time-dependent sensitivity and specificity evaluation. ROC curve summaries are based on ranking marker values in cases relative to controls and are thus unchanged by any monotone transformation of the data [20].

For landmark analysis, we chose time points *t* = 0, 1, 2, 3, 4, 5, and 6 years and estimate HR (*t*,*T*_final_) and AUC ^*C*/*D*^(*t*,*t*+1) at each of these time points. We also estimated both time-specific summaries, AUC ^*I*/*D*^(*t*) and HR (*t*), using non-parametric smoothing methods. Bandwidth selection for AUC ^*I*/*D*^(*t*) was done using cross-validation in order to obtain the bandwidth that minimized the integrated mean standard error for each marker. For HR (*t*), we used a fixed bandwidth of 0.3. We interpolated to estimate both AUC ^*I*/*D*^(*t*) and HR (*t*) at 6-month intervals, so that *t*=0,0.5,1,1.5,2,...,6 years.

We computed 95% bootstrap confidence intervals by resampling the data 200 times and obtaining percentile-based confidence intervals. Bias-corrected confidence intervals may also be calculated to adjust for finite-sample bias [26].

## Results

**Fig. 3:**
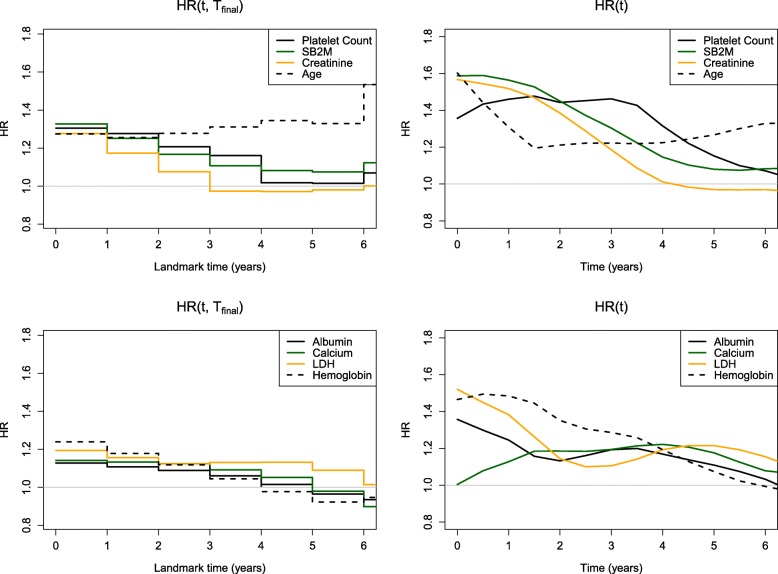
Marker performance over time using hazard ratios from landmark analysis and local linear estimation. The markers have been split up into two sets displayed in the top panel (platelet count, SB2M, creatinine, and age) and the bottom panel (albumin, calcium, LDH, and hemoglobin) for clarity. This figure appears in color in the electronic version of this article

**Fig. 4:**
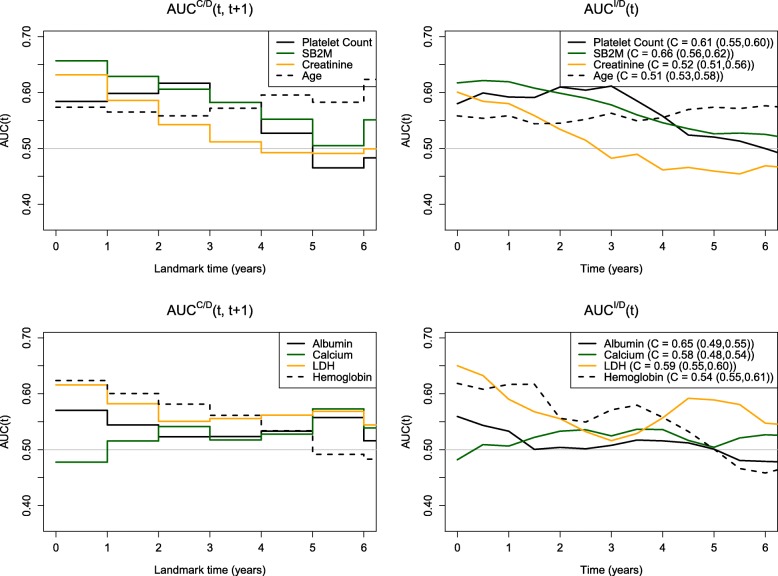
Marker performance over time using AUC ^*C*/*D*^ and AUC ^*I*/*D*^ (along with C-index estimates and corresponding 95% bootstrap confidence intervals). The markers have been split up into two sets displayed in the top panel (platelet count, SB2M, creatinine, and age) and the bottom panel (albumin, calcium, LDH, and hemoglobin) for clarity. This figure appears in color in the electronic version of this article

Figures 3 and 4 and Table 1 show comparisons of the four methods applied to the multiple myeloma dataset. In Fig. 3 (left panel), we see little separation in the landmark HR (*t*,*T*_final_) values between the different variables with initial hazard ratios approximately 1.30. All markers, with the exception of age, show a decline in performance over time. In Fig. 4 (left panel), we observe similar patterns across variables in terms of AUC ^*C*/*D*^(*t*,*t*+1) with qualitative similarity to the landmark results. Early values of AUC are approximately 0.60–0.65 but tend to decline toward 0.50 by year 5. The only inconsistency is in the performance of calcium, which appears to decline over time when assessed using HR (*t*,*T*_final_), whereas with AUC ^*C*/*D*^(*t*,*t*+1), we see an increase followed by a plateau. The right panel of Fig. 4 shows very close agreement between AUC ^*C*/*D*^(*t*,*t*+1) which defines cases cumulatively over 1-year intervals and AUC ^*I*/*D*^(*t*) which defines cases as incident events. Finally, HR (*t*) (Fig. 3, right panel) is more consistent with AUC ^*I*/*D*^(*t*).

**Table 1:** Comparison of summaries and rankings using HR (*t*,*T*_final_), HR (*t*), AUC ^*C*/*D*^(*t*,*t*+1), and AUC ^*I*/*D*^(*t*) at time points (a) *t*=1 year, (b) *t*=3 years, and (c) *t*=5 years

	HR(*t*,*T*_final_)			HR(*t*)			AUC ^C/D^(*t*,*t*+1)			AUC ^I/D^(*t*)		
	$\widehat {\text {HR}}$	95% CI	Rank	$\widehat {\text {HR}}$	95% CI	Rank	$\widehat {\text {AUC}}$	95% CI	Rank	$\widehat {\text {AUC}}$	95% CI	Rank
(a) *t*=1 year												
Platelet count	1.28	(1.16, 1.44)	1	1.46	(1.23, 1.75)	4	0.59	(0.53, 0.66)	3	0.59	(0.55, 0.63)	3
Albumin	1.08	(0.97, 1.21)	8	1.25	(1.07, 1.44)	7	0.52	(0.45, 0.57)	8	0.53	(0.49, 0.58)	7
SB2M	1.25	(1.15, 1.40)	3	1.56	(1.32, 1.84)	1	0.64	(0.57, 0.68)	1	0.62	(0.58, 0.66)	1
Calcium	1.15	(1.05, 1.33)	6	1.13	(0.98, 1.32)	8	0.54	(0.48, 0.61)	7	0.51	(0.47, 0.55)	8
Creatinine	1.17	(1.05, 1.32)	5	1.52	(1.33, 1.71)	2	0.58	(0.50, 0.64)	4	0.58	(0.54, 0.62)	5
LDH	1.14	(1.04, 1.26)	7	1.38	(1.22, 1.63)	5	0.56	(0.50, 0.61)	6	0.59	(0.55, 0.63)	4
Age	1.26	(1.15, 1.39)	2	1.31	(1.12, 1.50)	6	0.56	(0.50, 0.63)	5	0.56	(0.50, 0.59)	6
Hemoglobin	1.18	(1.08, 1.31)	4	1.48	(1.32, 1.71)	3	0.60	(0.55, 0.65)	2	0.62	(0.58, 0.65)	2
(b) *t*=3 years												
Platelet count	1.16	(1.01, 1.36)	2	1.46	(1.27, 1.72)	1	0.65	(0.57, 0.72)	1	0.61	(0.57, 0.65)	1
Albumin	1.08	(0.96, 1.31)	6	1.19	(1.05, 1.38)	6	0.54	(0.46, 0.61)	6	0.51	(0.46, 0.55)	7
SB2M	1.11	(0.97, 1.27)	4	1.30	(1.10, 1.49)	2	0.62	(0.53, 0.67)	2	0.58	(0.53, 0.61)	2
Calcium	1.09	(0.97, 1.29)	5	1.19	(1.04, 1.44)	6	0.48	(0.40, 0.57)	8	0.52	(0.50, 0.57)	5
Creatinine	0.97	(0.83, 1.11)	8	1.19	(1.00, 1.33)	6	0.52	(0.44, 0.58)	7	0.48	(0.44, 0.53)	8
LDH	1.14	(1.00, 1.30)	3	1.11	(0.99, 1.25)	8	0.54	(0.46, 0.61)	5	0.52	(0.50, 0.57)	6
Age	1.31	(1.16, 1.49)	1	1.22	(1.08, 1.40)	4	0.57	(0.50, 0.64)	4	0.56	(0.52, 0.59)	4
Hemoglobin	1.06	(0.94, 1.22)	7	1.29	(1.13, 1.47)	3	0.59	(0.51, 0.65)	3	0.57	(0.53, 0.61)	3
(c) *t*=5 years												
Platelet count	1.02	(0.85, 1.20)	4	1.15	(0.99, 1.38)	4	0.45	(0.35, 0.54)	8	0.52	(0.46, 0.54)	4
Albumin	0.98	(0.81, 1.23)	6	1.11	(0.95, 1.38)	5	0.56	(0.46, 0.66)	2	0.50	(0.45, 0.53)	6
SB2M	1.07	(0.91, 1.28)	3	1.08	(0.95, 1.23)	6	0.50	(0.39, 0.60)	4	0.53	(0.48, 0.56)	3
Calcium	1.00	(0.87, 1.40)	5	1.18	(1.05, 1.38)	3	0.61	(0.51, 0.71)	1	0.50	(0.50, 0.57)	6
Creatinine	0.98	(0.78, 1.18)	7	0.97	(0.83, 1.14)	8	0.48	(0.40, 0.57)	7	0.46	(0.42, 0.50)	8
LDH	1.11	(0.89, 1.33)	2	1.21	(1.04, 1.39)	2	0.56	(0.44, 0.64)	3	0.59	(0.53, 0.62)	1
Age	1.33	(1.13, 1.59)	1	1.27	(1.14, 1.47)	1	0.49	(0.41, 0.60)	6	0.57	(0.53, 0.60)	2
Hemoglobin	0.92	(0.77, 1.11)	8	1.07	(0.94, 1.24)	7	0.50	(0.38, 0.62)	5	0.50	(0.46, 0.54)	6

Compared to HR (*t*,*T*_final_), we see more non-monotonic trends across time for AUC ^*C*/*D*^(*t*,*t*+1), AUC ^*I*/*D*^(*t*), and HR (*t*). These results are not surprising, given that estimation of these measures is localized at each time point in contrast to the landmark HR summaries. For example, platelet count has relatively poor performance at baseline, peaks around 3 years, and continues to decline thereafter. In contrast, the time-specific trend gets averaged over follow-up time intervals by the landmark summary, HR (*t*,*T*_final_), and shows a steady decline in performance. As another example of HR (*t*,*T*_final_) flattening trends over time, observe that SB2M, creatinine, LDH, and hemoglobin have relatively good performance early on (HR (*t*)=1.38−1.56 at *t* = 1 year), which steadily declines over time (HR (*t*)=0.97−1.21 at *t* = 5 years). This trend is captured by all methods, except for HR (*t*,*T*_final_) (0.92−1.11 at *t* = 1 year versus 0.97−1.08 at *t* = 5 years).

In general, we see much better separation between the different markers using AUC ^*C*/*D*^(*t*,*t*+1), AUC ^*I*/*D*^(*t*), and HR (*t*), compared to HR (*t*,*T*_final_). A notable difference is seen between the hazard ratios of the left and right panels of Fig. 3. For example, at *t* = 1 year, the top and bottom ranking markers with respect to HR (*t*) have values of 1.56 and 1.13, respectively. In contrast, the top and bottom ranking markers with respect to HR (*t*,*T*_final_) have values of 1.28 and 1.08, respectively.

The rankings of the different variables are found to be fairly consistent across AUC ^*C*/*D*^(*t*,*t*+1), AUC ^*I*/*D*^(*t*), and HR (*t*). However, the estimates of the different summaries indicate that what may be considered fairly strong associations based on hazard ratios do not necessarily translate to good predictive ability as measured using AUC (*t*). For example, consider the top 4 ranking markers based on HR (*t*) at *t* = 1 year: SB2M, creatinine, hemoglobin, and platelet count, with statistically significant HR (*t*) values ranging from 1.46 to 1.56. A one-unit increase in each of these markers is associated with an added risk of approximately 50%. Meanwhile, the corresponding AUC ^*I*/*D*^(*t*) values range from 0.592 to 0.619, indicating poor predictive performance at 1 year.

Finally, our results are qualitatively different from those of [4], who concluded that SB2M retains good prognostic performance for all landmark time points, including later time points of 3, 4, 5, and 7 years. They dichotomized SB2M at 3.5 mg/L in their analysis while we analyze the biomarker in a continuous form after log-transforming and standardizing it. Our results using a continuous variable for SB2M show a HR (*t*) = 1.56 at 1 year versus 1.08 at 5 years and AUC ^*I*/*D*^(*t*) = 0.619 at 1 year versus 0.526 at 5 years. Using landmark analysis also yields weaker results with HR (*t*,*T*_final_) = 1.25 at 1 year versus 1.07 at 5 years.

## Discussion

We presented key summaries for evaluating the time-varying prognostic performance of a marker measured at baseline. A basic epidemiologic concept that distinguishes alternative summaries lies in the general idea of using cumulative versus incident events to define cases. Survival analysis using hazard models naturally focuses on incident cases. We found that the use of incident events naturally facilitates evaluation of time-varying performance either through the use of time-dependent hazard ratios or through time-dependent accuracy summaries. Comparing the two hazard ratio summaries, we found that local linear estimation of HR (*t*) revealed time trends more clearly given that it directly estimated the association at each time point *t*. In contrast, landmark analyses averaged across all time with *T*≥*t*, resulting in a time-averaged rather than time-specific association summary. Comparing the two time-dependent ROC curve summaries, we found that AUC ^*I*/*D*^(*t*) matched AUC ^*C*/*D*^(*t*,*t*+1) very closely; however, the latter used a coarser time scale. In the current descriptive context, hazard ratios obtained using local linear estimation and AUC ^*I*/*D*^(*t*) are potentially more desirable summaries compared to their landmark analysis counterparts. However, the sequential use of cumulative cases or landmark-based predictions may be useful in clinical settings where patient predictions are needed at select times.

Another key difference in the summaries was the use of hazard ratios from Cox regression versus ROC curves. A standard approach to analyzing survival data is to estimate hazard ratios. However, when the primary goal is to characterize prognostic performance, the question of interest may be more naturally addressed through approaches that quantify time-dependent classification error rates. Although our analysis showed similar patterns over time for time-varying hazard ratios and AUC ^*I*/*D*^(*t*), the latter has the advantage of being easy to interpret and compare across candidate markers measured on different scales. As discussed earlier, the hazard ratio is a measure of association and will depend on the scale of the marker, whereas time-dependent ROC curves quantify sensitivity and specificity. AUC ^*I*/*D*^(*t*) is a summary of these error rates and does not depend on the marker scale. Moreover, the hazard ratio does not lend itself to drawing clear conclusions regarding the strength of prognostic accuracy. While the AUC has a familiar and interpretable range of 0.5 to 1.0, it is unclear on the hazard ratio scale how large an association must be in order to indicate good prognostic performance. As has been noted by others [15], what constitutes a significant hazard ratio in studies of association does not necessarily reflect strong classification or prediction accuracy. For example, we saw with the multiple myeloma dataset that statistically significant hazard ratios of approximately 1.5 translated to AUC ^*I*/*D*^(t) values of only 0.6. Hazard ratios are also sensitive to the scale on which the marker is measured. Recall the marker transformations that were required in the multiple myeloma dataset in order to standardize markers so that their corresponding hazard ratios would be comparable. The ROC curve, on the other hand, remains unchanged by monotone transformations of the data. Finally, using available software, it is much faster to compute AUC ^*I*/*D*^(*t*) than it is to compute local linear estimates for hazard ratios and therefore calculation and comparison of AUC ^*I*/*D*^(t) for candidate markers can be performed for exploratory analyses.

A drawback of using ROC curves to summarize performance is that they handle ties in marker values poorly. This property can be problematic for categorical markers, where ties are highly prevalent. Given the common clinical practice of dichotomizing markers to classify patients into high- and low-risk groups, mishandling of ties can especially be an issue. For example, all of the markers studied by [4] were either inherently categorical (for example, performance status), or they were dichotomized versions of continuous markers. If a candidate marker is categorical or if a clinically established marker threshold is to be used to dichotomize the marker, regression methods are more appropriate than ROC curve methods for ranking candidate markers. However, if clinically established thresholds do not exist but are of interest, then ROC curves provide an avenue for exploring potential thresholds with the goal of optimizing the sensitivity and specificity.

We focused on markers measured only at baseline. However, the incident case ideas presented here can also be extended to longitudinal markers. A Cox regression model with time-varying covariates would handle a marker measured at multiple time points. Additionally, the incident/dynamic ROC curve can easily accommodate a time-varying marker (Bansal A, Heagerty PJ, Saha-Chaudhuri P, Liang CJ: Dynamic Placement Values: A Basis for Evaluating Prognostic Potential, unpublished).

Additionally, we focus on ROC curve methods for evaluating any single “biomarker,” which may commonly be the risk score derived from a model that includes multiple factors. The methods we discuss for evaluating a risk score in validation data are independent of those used initially for model selection in training data, in that they do not rely on the assumptions that may be necessary for the development of the risk score. One may use standard Cox regression or more flexible, modern machine-learning approaches for model development in training data. Regardless of the chosen modeling approach, the ultimate prognostic model is then fixed and used in the validation data to provide patient predictions of the disease outcome, i.e., a risk score.

Finally, our focus in this article is on evaluating a single prognostic marker or score or comparing individual candidate markers. Combining markers to improve performance is a related, but separate problem that we do not address here. We have previously published work aimed at establishing intuition about the expected incremental value under common, biologically motivated scenarios in the diagnostic setting with binary outcomes [2]. We expect similar results to hold in the current setting of prognostic markers with survival outcomes. However, as mentioned above, we assume here that if the time-varying performance of a multivariate risk score is being evaluated, optimal variable selection and model development have taken place prior to evaluation. The methods that we detail here can then be applied to any given risk score generated from a multivariate survival model. One of our findings from the multiple myeloma data analysis was that the AUCs for even the top ranking single markers did not exceed 0.7. This is not surprising for single markers, which often fail to have adequate performance on their own. In the development of multivariate prognostic scores, the question is often about evaluating the incremental value gained from a new marker when added to an existing baseline marker or model. The change in AUC is the most popular metric for evaluating incremental value. The time-varying AUC presented here can be used to evaluate the time-varying incremental value of a marker by estimating the time-varying AUCs of the baseline and enhanced models and taking their difference. Additionally, a number of alternative measures have been proposed in recent literature for binary outcomes, namely the net reclassification index (NRI) [18, 19] and integrated discrimination index (IDI) [19]. Extensions of these measures for time-dependent outcomes have been developed [7, 16, 22] and provide alternative summaries of the time-varying incremental value of a marker.

## Conclusions

It is common clinical practice to use the characteristics of a patient to predict his or her prognosis and in turn use such predictions to guide therapeutic decisions. Often, measurements from a single time point are used to guide decisions at multiple subsequent time points. However, predictive performance may vary over time. Accurately quantifying a marker’s time-varying performance would enable more informed decision-making. We illustrated alternative summaries and showed that although landmark-based predictions may be useful when patient predictions are needed at select times, a focus on incident events naturally facilitates evaluating trends in performance over time.

## Abbreviations

AUC: Area under the ROC curve
C/D: Cumulative/dynamic
C-index: Concordance index
FPR: False positive rate
HR: Hazard ratio
IDI: Integrated discrimination index
I/D: Incident/dynamic
K-M: Kaplan-Meier
LDH: Lactic hydrogenase
NRI: Net reclassification index
ROC: Receiver operating characteristic
SB2M: Serum beta-2-microglobulin
TPR: True positive rate
